# Is psychiatric emergency service (PES) use increasing over time?

**DOI:** 10.1186/1752-4458-3-3

**Published:** 2009-02-03

**Authors:** Michel Paradis, Carolyn Woogh, Dany Marcotte, Yves Chaput

**Affiliations:** 1 Department of Psychiatry, University of Montreal teaching hospital (CHUM), Montreal, Quebec, Canada; 2 Department of Psychiatry, Queen's, University, Kingston, Ontario, Canada; 3 Louis-H. Lafontaine Hospital, Montreal, Quebec, Canada; 4 365 rue Normand, Suite 230, Saint-Jean-sur-Richelieu, Quebec, J3A 1T6, Canada

## Abstract

**Background:**

Several recent studies have reported a significant increase in medical emergency department (ED) use for reasons of mental health. The diagnostic profile of these patients however differs from that usually described for patients visiting the psychiatric emergency service (PES). Few studies have specifically focused upon long-term PES utilization rates. Those that do typically present data from the early 80s, suggesting that deinstitutionalization may be an important contributing factor to the increases found. The aim of this study was to assess PES use using a more recent time frame and, the effects of non-specific factors, such as population growth, on this use.

**Methods:**

Visits per year at several different types of PESs were obtained; (a) for an 11-year period at a general hospital PES while the surrounding population remained stable, (b) at that same PES while the catchment area population doubled over a period of a few years, (c) for an 11-year period at two PESs without catchment areas while the surrounding population increased and (d-) for a 12-year period at a PES in a mental health facility while the surrounding population increased. Moderately conservative criteria were used to define either a trend or, a significant increase in utilization rates.

**Results:**

Each site had an inherent, 7 to 15% yearly variability in the number of PES visits. Over time however, only those where the surrounding population increased (either by an increase in the catchment area size or a regional increase in the population census) showed a trend or, a significant increase in utilization rates. These increases however were modest and of the order of 12 to 19%.

**Conclusion:**

Long observation periods are required in order to detect stable changes in PES utilization rates over time. As such, population growth may be but one of several factors underlying these increases. Organizational changes in mental health care delivery in the vicinity of the services that showed an increase could also have contributed. These latter would simply have redistributed (to the PES) the pre existing pool of mental health care patients, resulting in an increase that is more apparent than real.

## Background

A recent, nationwide "National Hospital Ambulatory Medical Care Survey (NHAMCS)" for the 1992 to 2001 period in the United-States reported a substantial increase (17.1 to 23.6 per 1000 population) in the number of medical emergency department (ED) visits for psychiatric-related reasons (PREDV) [1]. This rise was disproportionate in comparison to the overall rate of ED use during the same time period [1,2]. The diagnostic profile of PREDV patients is primarily weighted towards those with substance abuse, anxiety and mood disorders [1-3]. The reasons proposed for this increase in PREDV pertain to deficiencies in mental health care planning and financing such insurance issues, proportional reductions in mental health care expenditures, deinstitutionalization, reduced numbers of acute psychiatric care beds, as well as more clinical reasons such as increasing rates of mental illness or better detection at the ED level [1-6].

Increases in psychiatric emergency service (PES) utilization rates over time in the United-States have also been reported [7-9]. These data however are typically local (or regional) and emanate from the 70s and early 80s. Reasons such as deinstitutionalization, population growth, substance abuse and the development of the PES as a separate entity from the ED (154 PESs in 1963, over 2000 in 1984) are often cited as contributing factors [7-9]. The associated diagnostic profile is that of a young, chronic psychotic cohort, often co-morbid with a substance abuse disorder [7,8]. Later studies have not typically focused upon PES utilization rates. They have, however, supported the notion that patients with chronic or acute psychosis (schizophrenia/bipolar disorder/psychosis of unknown origin), substance abuse and personality disorders constitute a large part of the typical PES diagnostic profile [10-14]. Studies from the 1990s onward reporting (or where this data can be ascertained) on annual rates of PES use have not shown significant increases [15,16]. Generalizing from these studies however is difficult as they typically present data from a single-site, use relatively short (6-year) observation periods and factors such as variations in the surrounding population census are not reported.

The purpose of this study was to examine long-term (≥ 11 years) utilization rates of several different types of PESs (within general hospitals and in a psychiatric facility) through different time periods and under different population census conditions. The latter conditions included PES use while the surrounding population remained stable and while it gradually increased throughout the observation period. Lastly, PES use was assessed while the surrounding population rapidly doubled due to regional changes in mental health care delivery.

## Methods

All PESs in this study were in Canada and operated under a universal health care system. Two were in the city of Montreal, where each PES possesses a strict catchment area obliging citizens within it to seek acute psychiatric care at that PES only. Catchment areas are defined geographically. As such, the population within them can mirror changes in the overall city census. From 1986 to 1996 there was a slight (-2%) decline in the Montreal city population, which recovered between 1996 and 2001 (+2.3%) and increased minimally thereafter by 2.3% by 2006 [17].

Yearly visits to site 'A', the PES of a downtown Montreal university teaching hospital were obtained from June 15, 1985, to June 15, 2002. This site, whose characteristics have been described elsewhere [11], was in a general hospital and had medical triage prior to a psychiatric referral. It is a self-contained, secure unit with short-stay observation beds and is separate from the ED. Its catchment area remained stable from June 1985 to December 1996. After this date (between 1997 and 2000) the catchment area and the catchment area population doubled (to 180,000) due to the closing of a neighboring PES.

Yearly visit data from a second PES, site 'B', 9 kilometers east of site 'A', were available for the period between 1995 and 2006. Site B is the largest PES in the city and is located in a psychiatric facility. As such, it has no prior medical triage and operates as a 'walk-in' clinic. It is, however, also a self-contained and secure unit possessing short-term observation beds. It's geographic catchment area remained relatively stable throughout the observation period.

Data was also obtained from PESs in the city of Kingston. Kingston (city and metropolitan area) is approximately 300 Km west of Montreal, in the province of Ontario. PES referrals to the two Kingston general hospitals (1995 to 2005), the only sources of acute psychiatric care, were obtained (combined as site 'C') [18]. Neither PES has a catchment area, as is the case in Montreal. Between 1991 and 2006, the Kingston metropolitan area population increased by over 10% and the city proper had a 4% population increase from 1996 to 2006 [17].

### Primary data analysis

Yearly data per PES was averaged and standard deviations calculated (using Stata, Version 10). Moderately conservative criteria were used to define an increase in PES use in order to guard against false positive results. Also, it was assumed that year-to-year variations in the number of PES visits at each individual site might normally exist. A 'trend' required an increasing number of visits per year for at least 3 of the last 5 years of an observation period. Each of these three years must have a value greater than the mean for the total observation period at that site. Also, in one of the three years, the value must be equal to (or greater) than one standard deviation away from the site mean. If two of the three years met the last criteria, the trend was considered significant. This study does not include patient identification information it was exempted from full IRB review.

## Results

PES sites, some of their characteristics as well as some notable changes in mental health care delivery during the various observation periods are summarized in Table 1. The average number of visits per year at site 'A' for the eleven-year period when the catchment and city populations remained stable (June 1985 to June 1996) was 1,835 ± 109. No discernable increase in the number of PES visits per year could be observed during this time period (Table 2). Between 1997 and 2000 there was a progressive doubling in the number of citizens served by this PES. In the last year (June 2001 to June 2002) of the seventeen-year data acquisition period there were slightly over 3,600 PES visits. Between June 1996 and June 2002 the average number of visits per year increased to 2945 ± 751. During this time the Montreal city population increased slightly by about 2%, regaining the number of citizens lost between 1985 and 1996 [17].

**Table 1:** Site characteristics and some notable changes in mental health care delivery (CMHCD) during the observation periods.

Site	City location	PES location	Catchment area	Regular staff	Observation period^1^	CMHCD
A	Montreal	General Hospital	Yes	Yes	1985 – 2002	Yes^2^
B	Montreal	Psychiatric Facility	Yes	Yes	1995 – 2006	Yes^3^
C*	Kingston	General Hospital	No	Yes	1995 – 2005	Yes^4^

* Site 'C' comprises 2 PESs located in separate general hospitals. The same staff covered both sites.

^1 ^The yearly observation period at site 'A' began in June whereas it began in January at sites 'B' and 'C'.

^2 ^The closure of a neighboring PES, an increase in PES observation beds from 8 to 16, the opening of an outpatient day hospital.

^3 ^The closing of a PES approximately 10 km away.

^4 ^The gradual downsizing of a local psychiatric facility, a 30% reduction in acute general hospital psychiatric beds, the addition of specialized social workers in the PES both for the day and evening shifts.

**Table 2:** Raw yearly visit data for the participating sites^1,2^.

Site	1^st ^year	2^nd ^year	3^rd ^year	4^th ^year	5^th ^year	6^th ^year	7^th ^year	8^th ^year	9^th ^year	10^th ^year	11^th ^year	12^th ^year
A	1886	1974	1982	1927	1824	1828	1770	1636	1703	***1881***	1777	
B	4111	4239	3759	3697	4100	4179	4399	***4460***	***4795***	4098	***4474***	***4237***
C	1294	1210	1067	1217	1297	1450	***1382***	1313	***1360***	***1555***	***1512***	

^1 ^Cells in ***bold/italic ***are those that, during the last five years, are over the mean for the data acquisition period. Those in ***bold/italic/underlined ***are at least one standard deviation above the mean.

^2 ^The different time frames of the three observation periods are detailed in Table 1.

As there was a substantial change in the catchment area at site 'A' from 1996 onward we assessed the number of yearly visits at a site with a stable catchment area (site 'B') near site 'A', between 1995 and 2006. The average number of visits per year during the twelve-year observation period was 4212 ± 304 and, there was a trend suggesting an increasing number of visits per year during the final five years of the observation period (Table 2). From 1996 to 2006 the city of Montreal population increased by about 4.6%.

The Kingston metropolitan area benefitted from a more substantial population increase (over 10%) between 1991 and 2006 and the two Kingston city general hospital PESs (combined as site 'C') do not possess catchment areas. Their average number of yearly visits was 1332 ± 142 (11-year period from 1995 to 2005). These sites showed the largest increase in yearly numbers of PES visits (Table 2). Two of the four years (of the last five years) showed an increase above the mean that was at least one standard deviation or more.

## Discussion

Each individual PES in this study had an inherent 7 to 15% fluctuation (plus or minus) in the number of yearly PES visits over time. This suggests that prolonged observation periods are required when attempting to assess changes in PES use, especially in single-site studies. The present study used moderately conservative criteria to define these changes. Of the three PES sites reported on here, one was tagged as having a 'trend' and another, a 'significant' increase in use over time. Comparing the averages of the final five years with that of the first four at each of the two individual sites where an increase was found showed that they had a 12% and 19% increase in use, respectively. These values are quite modest compared to the reported 38% increase in PREDVs over a similar time period [1]. Given each site's inherent year-to-year variability care must be taken in interpreting our results.

Algorithms can be developed to detect changes in PES use over time at the national, regional and local level. Interpreting the significance of these changes however can be quite a challenge. Catalano et al., [19] reported that a host of factors, some clinical (both directly and indirectly related to the PES), some administrative and even, some environmental in nature, can influence weekly PES admissions. The authors concluded that a PES is both 'inextricably' tied to the mental health system and to 'events in the community it serves'. In the present study we examined but a single factor, population growth, on PES use. The increase at site 'B' was at a time during which the city population increased by 4.6%. Although site 'C' had the most robust increase, there was an over 10% increase in the Kingston metropolitan population between 1991 and 2006. In agreement with prior studies [15,16], yearly PES visits at site 'A' were unchanged during the time the surrounding population was stable.

A population influx over a prolonged period may disproportionately affect the PES for several reasons. For example, the longer the observation period the greater the proportion of visits made by a relatively small number of patients, primarily those with schizophrenia [7,11], which has a relatively stable prevalence rate in the population. Patients with schizophrenia (compared to those in the general population) suffer from greater medical co-morbidity, often due to conditions attributable to modifiable behaviors which in turn, can lead to greater psychiatric pathology [20,21]. Relatively small increases in their number via an increase in the population size could have a disproportionate effect on PES use. Noteworthy is that although chronic psychotic disorders, compared to anxiety disorders, represent a relatively modest proportion of the PREDV phenomenon, two of three PREDV studies report that they are the fastest growing sub-category in terms of percentages [2,22].

Long observation periods also highlight a host of other reasons, some of which are administrative in nature, that could at least partially account for the two relatively modest rises in PES use found in this study. Changes in mental health care delivery were observed in the vicinity of both sites. For site 'B, these included the closing of a PES approximately 10 Km away. The lack of prior medical triage at site 'B' (it operates as a 'walk-in' clinic) may have compounded this factor. Previous studies in Montreal [23] as well as in other regions of the world [24,25] have suggested that, at least with regards the ED, convenience of use and proximity can be factors contributing to a visit. In a prior PES study a higher percentage of visits judged neither pertinent nor urgent were observed at a PES located in a second psychiatric facility in the city of Montreal, compared to three PESs with prior medical triage located in general hospitals in this same city [26]. For site 'C', the progressive downsizing of the local psychiatric facility as well as a reduction in the number of acute psychiatric care beds (of approximately 30%) during the observation period could certainly have contributed to the overall increase in use.

In contrast, short-term population influxes appeared to correlate quite well with increased PES use. Doubling the catchment area population approximately doubled the number of yearly visits at the end of the observation period at site 'A'. Obviously, such a local increase in PES use would not have been detected at the regional (or national) level where aggregate or summary, rather than site-specific statistics, are used.

## Conclusion

Have PES visits been increasing over the last twenty years? The answer appears to be both yes and no, depending on how one defines the 'increase'. No PREDV or PES (including the present one) data from this period suggests that increases, when found, are primarily attributable to a true rise in the yearly number of *de novo *mental health cases. Rather, a host of administrative and clinical reasons may have resulted in an overall shift (towards the ED and the PES), of a largely pre existing pool of mental health patients. In addition, the PES may be sensitive to changes in the surrounding population, although the relationship between population growth and PES use over the long term may not be linear.

The above suggests that data acquisition at the local or regional level (versus the national or international level), may be particularly useful in understanding the complex intermingling of factors underlying variations in PES use. They also suggest that a fundamental understanding of local or regional mental health care delivery can be of substantial benefit when attempting to solve problems such as PES overcrowding.

## Abbreviations

PES: Psychiatric emergency service; ED: Medical emergency department; PREDV: Psychiatrically-related emergency department visit.

## Competing interests

The authors declare that they have no competing interests.

## Authors' contributions

YC was primarily responsible for data acquisition at site 'A' (in Montreal, Quebec), data analysis and, writing of the manuscript. CW was responsible for data at site 'C' (the two sites in Kingston, Ontario). DM was responsible for data at site 'B', in Montreal, Quebec. MP was also responsible for data at site A, in Montreal, Quebec.
